# Remembering to Forget: A Dual Role for Sleep Oscillations in Memory Consolidation and Forgetting

**DOI:** 10.3389/fncel.2019.00071

**Published:** 2019-03-12

**Authors:** Jesse J. Langille

**Affiliations:** Department of Neurology and Neurosurgery, McGill University, Montreal, QC, Canada

**Keywords:** cellular, synaptic, learning, NREM, REM, sharp wave, sleep spindle

## Abstract

It has been known since the time of patient H. M. and Karl Lashley’s equipotentiality studies that the hippocampus and cortex serve mnestic functions. Current memory models maintain that these two brain structures accomplish unique, but interactive, memory functions. Specifically, most modeling suggests that memories are rapidly acquired during waking experience by the hippocampus, before being later consolidated into the cortex for long-term storage. Sleep has been shown to be critical for the transfer and consolidation of memories in the cortex. Like memory consolidation, a role for sleep in adaptive forgetting has both historical precedent, as Francis Crick suggested in 1983 that sleep was for “reverse-learning,” and recent empirical support. In this article I review the evidence indicating that the same brain activity involved in sleep replay associated memory consolidation is responsible for sleep-dependent forgetting. In reviewing the literature, it became clear that both a cellular mechanism for systems consolidation and an agreed upon general, as well as cellular, mechanism for sleep-dependent forgetting is seldom discussed or is lacking. I advocate here for a candidate cellular systems consolidation mechanism wherein changes in calcium kinetics and the activation of consolidative signaling cascades arise from the triple phase locking of non-rapid eye movement sleep (NREMS) slow oscillation, sleep spindle and sharp-wave ripple rhythms. I go on to speculatively consider several sleep stage specific forgetting mechanisms and conclude by discussing a notional function of NREM-rapid eye movement sleep (REMS) cycling. The discussed model argues that the cyclical organization of sleep functions to first lay down and edit and then stabilize and integrate engrams. All things considered, it is increasingly clear that hallmark sleep stage rhythms, including several NREMS oscillations and the REMS hippocampal theta rhythm, serve the dual function of enabling simultaneous memory consolidation and adaptive forgetting. Specifically, the same sleep rhythms that consolidate new memories, in the cortex and hippocampus, simultaneously organize the adaptive forgetting of older memories in these brain regions.

## Introduction

Each evening we fall into an offline state defined by a diminished responsiveness to the environment, attenuated movement, intrinsically organized brain activity and bizarre thought patterns. It is during these sleep epochs that brains stabilize important, and erase unnecessary, information gleamed from waking experience in an attempt to ensure adaptive future behavior and efficient management of neural resources.

What follows is a general overview of sleep, oscillations, synaptic plasticity, neuromodulation, known interactions between these processes, memory consolidation and forgetting. Following the general overview of relevant topics, the remainder of this article is dedicated to reviewing what is known about the link between non-rapid eye movement (NREM) and rapid eye movement sleep (REMS) oscillations, memory consolidation and forgetting. As well as, advocating for a candidate cellular mechanism of sleep-dependent memory consolidation, speculating on potential sleep-dependent forgetting mechanisms and discussing a largely notional model for NREM-REMS cycling wherein sequential sleep stages serve to first lay down and edit and then stabilize and integrate engrams. Taken together, the aim of this article is three-fold: (I) to convince the reader that sleep oscillations act and interact in a coordinated manner to activate windows of synaptic plasticity; (II) to promote the notion that sleep oscillations, by activating transient windows of synaptic plasticity, mediate bi-directional memory processing; and (III) to advance a primarily notional model for NREM-REMS cycling compatible with objectives I and II.

### Sleep

Sleep is irresistible, necessary for survival and neurobiologically complex. Sleeps heterogeneous architecture can be deconstructed into NREM and REMS stages occupying 80% and 15%–20% of the offline period, respectively. NREMS is defined by a high-voltage, low frequency, synchronous electroencephalographic (EEG) pattern, vague, disconnected, mundane mentation and parasympathetic dominance (low heart rate, slowed breathing; Cabiddu et al., 2012; Carley and Farabi, 2016). Neuroanatomically, brain activity is near globally dampened during NREMS with stage prototypical neuronal activity programs arising from neocortical, thalamic and hippocampal neurons (Staresina et al., 2015). Comparatively, REMS is characterized by a wake like low-voltage, high frequency, de-synchronous EEG readout, vivid emotional mentation, muscular atonia and sympathetic dominance of peripheral physiological processes (elevated heart rate and ventilation). Neuroanatomically, REMS is distinguished from NREMS and wakefulness by strong activation of midline structures, collectively termed the REMS activation area, including the amygdala, pontine nuclei (Peever and Fuller, 2017) and the temporo-parieto-occipital junction, a region believed to be important for dream imagery. REMS also involves a hypoactivation of the frontal lobes (Nir and Tononi, 2010) and theta rhythmicity of hippocampal neurons (Spoormaker et al., 2013).

The ubiquitous nature of sleep in the animal kingdom indicates a robustly preserved biological process. Yet, a concise and accepted core function of sleep has yet to be elucidated. Proposed functions of NREMS include: (I) brain waste clearance by the perivascular glymphatic system (Plog and Nedergaard, 2018); (II) cellular prophylaxis and restoration (Vyazovskiy and Harris, 2013), accomplished by the window of decreased electrophysiological activity/metabolic demands (Van Cauter et al., 1997; Sharma and Kavuru, 2010) and heightened growth hormone secretion (Van Cauter and Plat, 1996) provided by NREMS; (III) systems level memory consolidation (Hardt and Nadel, 2018); this process is discussed in detail in the “Consolidation” section below; (IV) gist extraction, which functions to remove noisy, irrelevant information while preserving important data (Feld and Born, 2017); and (V) synaptic homeostasis, a process organized to renormalize net synaptic strengthen to some baseline following its elevation during waking (Tononi and Cirelli, 2014; Lewis et al., 2018).

Proposed functions of REMS include: (I) aqueous humor distribution (Modarreszadeh et al., 2014). Specifically, ocular movements during REMS may stir up eye fluids to provide nutrients and oxygen to the avascular cornea; (II) an ontogenetic/developmental hypothesis (Marks et al., 1995) suggests that brain activity during REMS aids in connection formation. The ontogenetic hypothesis is compatible with research indicating that REMS is most prevalent during development and decreases with age (Skeldon et al., 2016); (III) the restoration of monoaminergic receptors (Rotenberg, 2006), perhaps accomplished by the period of low monoaminergic tone provided during REMS; (IV) simulation and subsequent habituation of emotional scenes (Cunningham et al., 2014); (V) emotional memory consolidation (Hutchison and Rathore, 2015). Hypotheses IV and V align with the tendency for emotional brain areas such as the amygdala to be hyperactive during REMS; (VI) non-declarative memory consolidation (Cedernaes et al., 2016), a process which may benefit from the ability of procedural memories to be “rehearsed” without consequence during REMS owing to the atonia/paralysis of skeletal muscle (McCarter et al., 2013); (VII) synaptic consolidation, discussed below in the “Consolidation” section (Diekelmann and Born, 2010); (VIII) the integration of newly encoded information into existing information frameworks (Sterpenich et al., 2014); (IX) the formation of novel, non-intuitive connections capable of facilitating future divergent thought *via* abstraction and extrapolation (Cai et al., 2009; Lewis et al., 2018); (X) the “remembering” or stabilization of not yet consolidated memories; and (XI) the forgetting of previously consolidated memories (Poe, 2017). Hypotheses X and XI will be discussed in detail in sections: REMS Oscillations and Memory Consolidation and REMS Oscillations and Forgetting, respectively. Generally, sleep functions can be delegated to one of two categories: maintenance and repair or information processing. I focus here on the latter category, and in particular on how the sleeping brain accomplishes this information processing function. Which, appears to be by co-opting offline brain oscillations to simultaneously “download/save” important, and “delete/erase” noisy, un-adaptive information.

### Sleep Oscillations

While neuronal activity in the awake brain largely reflects the processing of information pertaining to the external environment, the electrical activity of the sleeping brain results from internally generated oscillatory patterns (Olbrich, 2010). Oscillations are rhythmic fluctuations in brain activity (Thut et al., 2012) and are crucial for sleeps function as an information processing state. During NREMS the cortex is dominated by low frequency, synchronous delta (0.5–3.5 Hz; Harmony, 2013) and slow waves (0.5–1 Hz; Bellesi et al., 2014), which are intermittently interrupted by several irregular waveforms. These irregular waveforms include sleep spindles (SSs; 11–15 Hz; Purcell et al., 2017), K-complexes (<1 Hz; Lucey, 2017) and sharp wave-ripples (SPW-Rs; 150–200 Hz; Liu et al., 2017).

Of the slower, more synchronous NREMS oscillations, delta waves are primarily generated in the thalamus (Crunelli et al., 2011) while slow waves arise predominately from periodic fluctuations in the membrane potential of cells in the superficial layers of the frontal cortex (Nir et al., 2011; Halgren et al., 2018). Delta and slow waves, sometimes collectively termed slow wave activity or slow oscillations (SOs), consist of alternating depolarizing “up” and hyperpolarizing “down” states (Neske, 2016). The depolarizing SO up state is believed to serve a memory function (Heib et al., 2013) and is the phase of the SO considered throughout much this article. Comparatively, the hyperpolarizing SO down state may provide a period of cellular “rest,” allowing for prophylaxis and restoration (Vyazovskiy and Harris, 2013).

SSs and K-complexes are generated by the thalamus and cortex (Amzica and Steriade, 1998; Mak-McCully et al., 2017). In comparison to the synchronous NREMS oscillations, the role of these irregular electrophysiological phenomena is less clear. SSs are often implicated in memory processing (Mednick et al., 2013; Schonauer, 2018) while K-complexes seem to be important for suppressing neuronal responses to external stimuli that the brain has deemed irrelevant and non-dangerous, ensuring consolidation of the rest phase (Peng et al., 2014). A suppressive function of K-complexes is supported by the finding that these waveforms are evoked by external stimuli (Forget et al., 2011). Further, K-complexes produce a strong hyperpolarization, observable as a large deflection on the EEG readout, well suited for rapidly suppressing cortical excitation (Cash et al., 2009). The other irregular NREMS waveform mentioned, the SPW-R, is comprised of two, interacting waveforms, the hippocampal CA3 sharp-wave (0.01–3 Hz) and the CA1 ripple (Csicsvari et al., 2000). SPW-Rs are considered to have a memory function (Buzsáki, 2015).

During REMS neuromodulation from REMS inducing pontine nuclei, namely the pedunculopontine and lateral dorsal tegmental nuclei, increases neuronal excitability (Ye et al., 2009; Van Dort et al., 2015). This increased neuronal excitability, when combined with the interconnectivity of the cerebral cortex, produces a fast, de-synchronous cortical activity readout. The hippocampus is spared from this de-synchrony and instead exhibits theta rhythmic activity during REMS (Brown et al., 2012); there is some debate as to whether REMS theta rhythm is intrinsic (Goutagny et al., 2009) or is a product of projections from the medial septum behaving as a pacemaker (Stewart and Fox, 1990). Functionally, REMSs cortical de-synchrony and hippocampal theta rhythm are involved in memory processing (Buzsáki, 2002; Sara, 2010). Those NREM and REMS oscillations described as being involved in memory exert said involvement by organizing the brains activity to produce windows in time where the conditions are optimal for synaptic plasticity, the cellular basis of memory processing.

### Synaptic Plasticity

Neurons possess the unique capacity to store information by modifying the strength of their interconnections. In his seminal work, Hebb (1949) suggested that it is the co-activation of neurons by experience that triggers some fundamental mechanism capable of changing the brains connections and, in the process, learning information from the environment. Specifically, Hebb postulated that when two neurons fire simultaneously the connection between them is strengthened, by some “cellular growth” or “metabolic change,” such that the activation of one cell is now more likely to spread and activate the other. The ability of neurons to modify their connection strengths is termed synaptic plasticity.

Since the time of Hebb much has been learned about the neurobiology of learning and memory (Brown and Milner, 2003; Fox and Stryker, 2017). It is now known that the timing of neuronal activations, particularly the relative timing of presynaptic glutamate release and postsynaptic spiking, determines the extent (and directionality, discussed below) of connection strength change. Activation timing controls the extent and directionality of plasticity by shaping N-methyl-D-aspartate (NMDA) channel open times and thus the magnitude of calcium influx (Feldman, 2012); kinases activated by high levels of dendritic calcium (protein kinase A) and phosphatases activated by low levels of dendritic calcium (calcineurin/protein phosphatase I) are key regulators of synaptic strengthening (Roberson and Sweatt, 1996) and synaptic weakening (Isaac, 2001), respectively (Castellani et al., 2005). The series of timing rules that shape synapse strength changes are sometimes termed Hebbian spike-timing-dependent plasticity (STDP), in honor of the contribution of Hebb’s theories to our understanding of the neurobiology of learning and memory.

Although not a focus of Hebb’s writings, it is now known that synaptic plasticity is a bi-directional process consisting of the synaptic efficacy augmenting long-term potentiation (LTP) and efficacy weakening long-term depression (LTD; Bear, 2003). At the cellular and molecular level synaptic plasticity involves changes in synapse structure and in the number and/or phosphorylation status of membrane channels. Increasing the number and size of synapses increases the total surface area connecting two neurons, while increasing the number of and/or phosphorylating receptors increases a neurons conductance potential (Citri and Malenka, 2008). LTP is believed to be the principal cellular mechanism by which neurons encode complex information gleamed from experience (Miller and Mayford, 1999; Langille and Brown, 2018). Comparatively, LTD seems to be important for a simpler form of learning, habituation (Glanzman, 2009). Habituation is the process by which an animal decreases their behavioral responsiveness to a repeatedly presented, innocuous stimulus (Rankin et al., 2009). All said, modern experimental research on synaptic plasticity has confirmed Hebb’s (1949) theory of learning.

Additional forms of synaptic plasticity not mentioned here include non-Hebbian and homeostatic plasticity. For more information, readers should consider the following detailed reviews (Turrigiano, 2012; Piochon et al., 2013).

### Sleep Oscillations and Synaptic Plasticity

Oscillations are fluctuations in membrane excitability. As eluded to in the previous section, changes in synapse strength typically require the activation/inactivation of membrane channels sensitive to voltage, such as the NMDA receptor. Therefore, oscillations have the capacity to alter the likelihood that various biochemical cascades, acting downstream of changes in membrane channel conductance and upstream of synaptic plasticity, are activated (Hölscher, 1999). As an example, moderate activation of a cortical synapse during the down state of a SO, when membrane excitability is low, may lead to a brief NMDA receptor open time, low magnitude calcium influx and LTD. Comparatively, the same moderate activation, of the same cortical synapse, during the SOs depolarizing up state may elicit sufficient excitation to cause long NMDA channel open times and high magnitude calcium influx, culminating in the maintenance of connection strength at the activated synapse. A relatively recent study conducted in humans provides support for the above example by demonstrating that SO up state duration is related to the magnitude of post-sleep memory performance enhancement (Heib et al., 2013). The authors of the study suggest that the correlation between SO up state duration and the degree of subsequent memory improvement results from changes in the amount of time available for the conversion of memory from recent to remote. Heib et al.’s (2013) work bolsters the above example as prolonged SO up states would cause NMDA channels to remain open longer and produce the high magnitude calcium influx necessary for memory stabilizing potentiation. However, recent research by González-Rueda et al. (2018) shows that both maintenance and depression of synapse strength can result from SO up states, adding an additional tier of complexity to the aforementioned example. These authors suggest that during the up states of NREMS SOs synapses participating in memory associated cell assemblies cooperate to aid in postsynapse firing and as a result maintain their synaptic strengths while synapses which do not assist in postsynapse firing are subjected to weakening (González-Rueda et al., 2018). Notably, dendrite sub-region specific subthreshold input cooperation (Lee et al., 2016) may contribute to the synaptic weight maintenance during SO up states. Taken together, these findings indicate that during sleep brain waves organize synaptic plasticity such that new or strong memories are safeguarded from, while weak memories are subjected to, SO up state driven synaptic downscaling (González-Rueda et al., 2018). As further exemplification of the relationship between oscillatory phase and plasticity, the REMS hippocampal theta rhythm is, like the SO, a sinusoidal waveform wherein the directionality of plastic change has been documented to depend on the phase (up vs. down state/limb) of the oscillation (Pavlides et al., 1988).

Oscillations may be necessary for synaptic plasticity during sleep. In support of this claim, Durkin et al. (2017) demonstrate that NREMS oscillations are crucial for the synaptic plasticity involved in transferring information captured by the visual thalamus during experience to the primary visual cortex. At least two reasons for the dependency of certain plastic changes on sleep oscillations can be levied. First, changes in neuromodulation during sleep can alter membrane channel conductance properties. Second, sleep associated changes in transcription can alter the level of intracellular and membrane proteins and thus the composition of the synaptic proteome (Timofeev and Chauvette, 2017). Both of these changes lead to shifts in membrane excitability and thus re-shape the regional activity patterns necessary for activating synaptic plasticity pathways. Sleep-dependent oscillations may be well suited to satisfy these altered plasticity requirements.

Oscillations do not occur in isolation (Steriade, 2006; Jensen and Colgin, 2007). By coupling specific oscillations, the brain can shape what synaptic plasticity pathways are facilitated, in what brain regions and at what times. In the earlier example activation of a synapse during the SO up state was described as producing a “maintenance” level of excitation, not weak enough to trigger LTD nor strong enough to trigger lasting LTP. However, if a SS is coupled to the SO up state the same activation can produce sufficient excitation to trigger consolidative plasticity (Kim S. Y. et al., 2017), such as LTP. These observations beg the question, why is it that during wakefulness information is readily encoded and during sleep precisely timed oscillatory interactions are necessary to trigger similar synaptic plasticity? The answer may lie in state specific differences in neuromodulation, discussed below.

### Neuromodulation Organizes Oscillations and Synaptic Plasticity

Both arousal state and synaptic plasticity are shaped by neuromodulators acting to produce shifts in membrane excitability (Lee and Dan, 2012; Nadim and Bucher, 2014; Palacios-Filardo and Mellor, 2019). Specifically, research by Ding et al. (2016) indicates that changes in the ionic composition of the brains extracellular milieu, shaped by neuromodulators acting on ion channels and transporters, is what shifts the brain through various arousal states by altering neuronal membrane excitability. During wakefulness, high cholinergic and monoaminergic (histamine, serotonin, dopamine and norepinephrine) neuromodulation increases membrane excitability and overall brain activity producing a neuronal environment conducive for potentiating plasticity (Vyazovskiy et al., 2009; Schwartz and Kilduff, 2015). During NREMS, neuromodulation dampens (Brown et al., 2012) and membrane excitability decreases producing an unfavorable environment for potentiating synaptic plasticity. Yet, NREMS has a well-documented role in memory consolidation (Cox et al., 2012; Rasch and Born, 2013), a process clearly involving synaptic strengthening. In order to reconcile these seemingly opposite qualities of NREMS one must consider that something other than neuromodulation is acting to gate synaptic plasticity during sleep. Coupled brain waves have been shown to organize cortical dynamics in favor of synaptic plasticity (Zarnadze et al., 2016) and may, through the use of mechanisms akin to those described in the previous section, offer a solution. In line with this suggestion, neural activity during sleep is primarily driven by intrinsically generated brain waves orchestrated by the principal neurotransmitters glutamate (Hughes et al., 2002) and gamma-aminobutyric acid (GABA; Sun et al., 2012). As an example, during wakefulness thalamo-cortical relay neurons are tonically depolarized by neuromodulation from the ascending reticular activating system, a diverse series of neuromodulatory neurons dispersed within phylogenetically older brain regions, including the brainstem (McCormick and Bal, 1997). During NREMS, the ascending reticular activating system is silenced and the membrane potential of thalamo-cortical relay neurons moves further from spike threshold. Freed from waking neuromodulation, these thalamo-cortical neurons assume their intrinsic, slow, oscillatory activity patterns driven by hyperpolarization activated ion channels on the neuronal membrane as well as by reciprocal interactions between the thalamus, cortex and GABAergic thalamic reticular nucleus (Brown et al., 2012). These state-dependent, neuromodulation driven changes in thalamo-cortical neuron membrane potential is what allows the thalamus to readily relay information from the environment during wake and to disconnect one’s mind from the outside world during sleep (Lam and Sherman, 2011; Sun et al., 2012).

During REMS, increases in cholinergic neuromodulation elicit increases in neuronal membrane excitability (Picciotto et al., 2012). REMSs increase in cortical membrane excitability, driven primarily by the modulation of potassium channel activity by acetylcholine (Gottesmann, 2002), produces wake like neural activity (Vakalopoulos, 2014) and an environment favorable for potentiating plasticity (Rasch and Born, 2013). In sum, during wakefulness and REMS neuromodulation primes the brains neurons for potentiating plasticity. While during NREMS, when excitation promoting neuromodulation is absent, interacting oscillations may replace the role of neuromodulation in the gating of synaptic plasticity.

### Consolidation

Information captured by the brain is inherently unstable upon initial encoding and ascends along a trajectory of increasing stability over time by moving through successive neurobiological embodiments. A declarative memory adopts three neurobiological embodiments, or traces, during its tenure: (I) reverberation (activity-dependent or working memory; Constantinidis and Klingberg, 2016; Riley and Constantinidis, 2016); (II) weighting plasticity engram; and (III) wiring plasticity engram.

Working memory is a transient form of information preservation dependent on the reverberation of neural circuits (Nyberg and Eriksson, 2015). Information held in working memory that is either salient, thoroughly processed or both may trigger stabilizing synaptic plasticity, crystallizing held information into activity-independent storage (Dubnau et al., 2003). Weighting plasticity describes the processes through which the hippocampus accomplishes the rapid, initial, limited capacity, interference susceptible, temporary, activity-independent storage of information (Cheng, 2013; Frankland et al., 2013; Preston and Eichenbaum, 2013). Engrams derived from weighting plasticity mechanisms are comprised exclusively of metabolic change (i.e., these engrams result from a re-weighting of existing synaptic connections; Frankland and Bontempi, 2005). Hippocampal anatomy enables rapid information encoding through weighting plasticity as a large portion of all possible inter-neuronal connections exist at any given time (Kali and Dayan, 2000) and can “simply” be differentially weighted to encode information (Takeuchi et al., 2014). The neocortex, by comparison, contains a paucity of all possible connections at any moment in time, owing to energetic, material and network stability constraints. Because of this feature of neocortical anatomy information storage in this region can use both weighting plasticity as well as slower, but more stable, wiring diagram changes to encode information activity-independently (Lisman and Morris, 2001). These wiring changes, which include alterations in synapse morphology and or the number of synapses, are produced through the mechanisms of wiring plasticity (Chklovskii et al., 2004; Holtmaat and Svoboda, 2009). In sum, hippocampal weighting plasticity enables the rapid, temporarily sustained capture of reverberating information while wiring plasticity, in the vast circuits of the cortex, affords a stable, interference resistant, quasi-infinite capacity, long-term storage of information (Alonso et al., 2005; Mednick et al., 2011).

Memory consolidation is the term given to the processes used by brains to move information through the described embodiment or trace succession. These consolidative processes stabilize important information gathered during waking so that it can be used to adaptively guide future behavior. Memory consolidation can be synaptic or systems level and online or offline. Synaptic consolidation describes the biochemical and biophysical stabilization of reverberating information into an activity-independent engram (Diekelmann and Born, 2010). Systems consolidation describes the time protracted process through which information moves from the hippocampus to the cerebral cortex (Born and Wilhelm, 2012; Hardt and Nadel, 2018). Both synaptic and systems consolidation can occur on or offline. Though, it can be argued that synaptic consolidation is favored while an animal is online and systems consolidation is favored once the animal goes offline (Gais and Born, 2004a,b). Mechanisms for offline systems and synaptic consolidation are described below; see “NREM Oscillations and Memory Consolidation” as well as “REMS Oscillations and Memory Consolidation” sections.

Importantly, not all information captured by the brain undergoes the consolidative transcendence from a reverberation to a weighting plasticity engram or from a weighting plasticity engram to a wiring plasticity engram. Information that is not particularly salient or processed meaningfully in working memory, along with information that is encoded in weaker forms of LTP, rapidly deteriorates (Frey et al., 1993; Raye et al., 2002; Curtis and D’Esposito, 2003), often before consolidative processes occur. This kind of “passive” or “natural” forgetting differs from the “active” or “sleep-dependent” forgetting discussed below.

### Forgetting

If all the information an animal encountered was retained, and forever, the brains circuits, material limitations, energetic capacities and the ratio of adaptive to irrelevant, non-adaptive information (herein termed the signal to noise ratio) would quickly become saturated. Such saturation would prevent the useful application of acquired knowledge and diminish any capacity for future learning. For these reasons, information stored by the brain needs to be regularly and selectively cleared. The clearance of information, characterized by a decreased accessibility and/or fidelity of said information and termed forgetting, is therefore crucial for healthy brain function, adaptive behavior and learning.

Forgetting can occur in an active or passive manner. Active forgetting is deliberate, organized and acts to selectively clear unnecessary information from the brain. Comparatively, passive forgetting is unintentional and disadvantageous, often causing adaptive information to become decreasingly available and veridical. Notably, the active forgetting described here refers to a series of adaptive, sleep-dependent processes organized for gist extraction and the clearance of unimportant memories and differs from other forms of active forgetting described elsewhere, including intrinsic and motivated forgetting (Davis and Zhong, 2018).

As an example of active forgetting during sleep, Saletin et al. (2011) demonstrate that pre-sleep instruction influences which memories are stabilized or forgotten during a subsequent sleep session. Specifically, the authors found that memories participants were instructed to forget did not experience the same benefit from sleep that memories participants were told to remember did. These findings support the thesis that sleep can carry out forgetting in an active and organized manner, as opposed to simply resulting from global, passive, unintentional clearance operations.

As described earlier, the brain stores information through weighting (post-translational modifications and receptor trafficking; Hardt et al., 2013) and wiring (growth of existing and activation/formation of new synapses; Kandel et al., 2014) plasticity. Neurobiologically, forgetting results from the disintegration of these changes. During forgetting, phosphatases, including calcineurin and protein phosphatase-1 (Sachser et al., 2016), remove post-translational marks while the Rac1/Cofilin pathway allows for the disassembling of structural changes (Davis and Zhong, 2018).

There are at least eight brain mechanisms capable of triggering the neurobiological change associated with forgetting: (I) neurogenesis-dependent circuit rearrangement/overwriting (Gao et al., 2018); (II) pro-active interference (Alves and Bueno, 2017); (III) retro-active interference (Alves and Bueno, 2017); (IV) engram instability (transience of weighting plasticity; Davis and Zhong, 2018); (V) sharp wave replay-induced depotentiation (Norimoto et al., 2018); (VI) homeostatic synaptic downscaling (Tononi and Cirelli, 2014); (VII) theta trough replay (Poe, 2017); and (VIII) low frequency oscillation evoked depotentiation (Tononi and Cirelli, 2006); a summary of these forgetting mechanisms is included as Table 1.

**Table 1:** Neurobiological mechanisms of forgetting.

	**Sleep independent mechanisms**
Neurogenesis-dependent overwriting	The incorporation of new born neurons into hippocampal circuitries re-organizes existing connections and over-writes information stored in previously formed wiring diagrams. Neurogenesis associated re-wiring may gradually decrease the size and fidelity of, as well as accessibility to, engrams
Pro-active interference	Older information represented in stabilized wiring diagrams can impede the recall or storage of new information
Retro-active interference	Old information can be over-written by new information recruiting similar circuitries, due to a reallocating of cellular materials (biochemical and circuit resources)
Engram instability	Information stored as metabolic change is inherently unstable as biochemical changes are subject to regular turnover and degradation
	**Sleep dependent mechanisms**
Sharp wave replay	Select information replayed in sharp-waves weakens as these oscillations produce presynaptic and postsynaptic decoupling and engage depotentiation pathways
Homeostatic synaptic downscaling	Sleep sees a proportional downscaling of synaptic weights aimed at preventing run-away potentiation and resource exhaustion. This downscaling operates through a biochemical mechanism involving Homerla. Though, electrophysiologically evoked mechanisms are also involved; see row 8
Theta trough replay	Information replayed during periods of decreased neuronal excitability (theta troughs) engages depotentiation or LTD-like pathways
Low frequency oscillation evoked depotentiation	Low frequency oscillations can decouple synaptic activity and evoke biochemical cascades that disintegrate engrams

*Shown are brief descriptions of several sleep independent (rows 1–4) and sleep-dependent (rows 5–8) forgetting mechanisms*.

Of the forgetting mechanisms described above, the latter four are sleep-dependent. These sleep-dependent forgetting mechanisms can be realized by the same oscillations known to be important for sleep-dependent memory consolidation: hippocampal sharp-waves (V), cortical SOs (VI and VIII) and hippocampal theta rhythm (VII), as discussed below. Notably, mechanisms VI and VIII are similar in that both can be realized by SOs. The distinction between the two is that homeostatic synaptic downscaling can also arise from mechanisms which are at their core biochemical (Maret et al., 2007; Siddoway et al., 2014; Diering et al., 2017) and independent of network activity during sleep. The four sleep-independent mechanisms listed above, I, II, III and IV presumably also operate during sleep.

## NREMS Oscillations and Memory Consolidation

Learning during wakefulness changes the brains activity patterns during sleep, largely in favor of memory processing. The SPW-R waveform, when observed during sleep, reflects the offline, temporally-compressed replay of neuronal sequences active during recent, online learning (Atherton et al., 2015). These rhythms increase markedly in the sleep episode following associative learning, relative to other forms of learning or recall (Eschenko et al., 2008), and improve subsequent performance (Ramadan et al., 2009). Accordingly, disrupting SPW-Rs impairs later performance (Girardeau et al., 2009) and new learning (Norimoto et al., 2018). The memory improvement observed following increases in SPW-Rs comes from the involvement of these oscillations in the inter-regional communication component of memory consolidation (Buzsáki, 2015). Other NREMS oscillations, including neocortical SOs (Marshall et al., 2006), thalamic SSs (McDevitt et al., 2017) and cortical ripples (Khodagholy et al., 2017) have also been implicated in this veridical preservation process.

Like SPW-Rs, SOs and SSs are more numerous in the sleep that follows learning, and act to improve memory processing and performance. More specifically, after learning SO depolarizations increase while down states modify (Mölle et al., 2009). Marshall et al. (2006) and Schneider et al. (2015) demonstrate that augmenting SOs by transcranial stimulation improves memory retention on paired-associate and picture recognition learning tasks. SSs occur rhythmically and, in addition to becoming more numerous following learning (Gais et al., 2002; Morin et al., 2008), increase after exposure to memory cues (Antony et al., 2018; Cairney et al., 2018). In Antony et al.’s (2018) study, to improve memory cues were presented to sleeping subjects immediately following the inter-spindle period, when new spindles ought to be occurring. The authors suggest that the rhythmic organization of spindles, into active and refractory (inter-spindle) periods, may serve to deliberately separate memory reactivations in time; how SSs regulate memory reactivations is discussed below. Structuring memory reactivations in this way would help preserve memory fidelity by preventing overlap or undesired integration of reactivated memories, resulting from coincident or lingering plasticity pathway activation. In another study, increases in SS frequency at the same time as cued-memory reactivation were found to be correlated with improved performance on a motor learning task (Laventure et al., 2016).

In regards to ripples, Khodagholy et al. (2017) found that the coupling of hippocampus and association cortex ripples is beneficial for systems consolidation. Similarly, Axmacher et al. (2008) found a correlation between ripples in rhinal cortices and performance on an item recall task learned prior to sleep, when administered again after waking. Thus, SPW-Rs reactivate memories while other NREMS oscillations, including SOs and SSs, are auxiliary to these memory reactivations and facilitate memory processing. As discussed below, these auxiliary oscillations organize windows in time during which information is transferred and engraved into cortical circuits.

Cortical SOs and thalamic SSs cannot consolidate memories independently as they do not have access to recent memories stored in the hippocampus. Yet, SOs and SSs, like SPW-Rs, increase following learning (Gais et al., 2002; Eschenko et al., 2008; Morin et al., 2008) and improve memory (Marshall et al., 2006; Ramadan et al., 2009; Kaestner et al., 2013). These conflicting observations are explained by an interactive model wherein NREMS oscillations couple and this coupling mediates memory consolidation (Staresina et al., 2015). Memories formed in the auto-associative circuitry of the CA3 field spontaneously reactivate as SPWs and influence CA1 ripples, which nest in the troughs of SSs triggered by SOs (Csicsvari et al., 2000; Staresina et al., 2015; Latchoumane et al., 2017). By triggering SSs SOs ensure that the memory reactivations (SPW-Rs) carried in spindle troughs reach the cortex during the depolarizing phase of the SO. Bergmann et al. (2012) have shown that SS activity is highly correlated with hippocampal BOLD signal and that this pairing is concomitant with activity at distinct neuroanatomic loci involved in category-specific prior learnings. An interactive model is compatible with the studies mentioned above, which demonstrate the influence of learning on sleep oscillations and memory performance. Specifically, after learning an increased number of SOs and SSs would have more opportunities to couple or transmit SPW-Rs, resulting in enhanced consolidation and improved performance. In short, during sleep a systems consolidative dialog between memory encoding (hippocampus) and long-term storage (cerebral cortex) structures manifests as an interregional, cross-frequency, EEG signature. Namely, SO up states coupled to spindle trough nested ripples.

Offline, SPW-R memory reactivations couple with SOs and/or SSs to mediate systems consolidation (Mölle and Born, 2011; Staresina et al., 2015; Latchoumane et al., 2017). However, online, isolated SPW-Rs have also been observed (O’Neill et al., 2006) and proposed to, amongst other things, consolidate memory (Joo and Frank, 2018). However, several lines of evidence suggest that these waking SPW-Rs may be insufficient for meaningful consolidation:

I. If waking SPW-Rs were sufficient for consolidation an increased duration of waking, i.e., sleep restriction, should improve memory performance, the opposite of which is observed (Kamphuis et al., 2017);
II. During wakefulness an acetylcholine rich neurochemical milieu prohibits SPW-R mediated hippocampal-neocortical dialog (Vandecasteele et al., 2014);
III. SPWs, absent SOs and SSs, may cause synaptic weakening through engagement of the decoupling force of STDP (Norimoto et al., 2018);
IV. Online consolidative memory replay would be biased by immediate waking experiences. Comparatively, during sleep a brains knowledge can be sampled in an unbiased way (Tononi and Cirelli, 2014);
V. SPW-Rs in awake animals typically occur during exploratory pauses and have been suggested to have a function in trajectory planning and memory retrieval (Gupta et al., 2010; Carr and Frank, 2012; Pfeiffer and Foster, 2013; Joo and Frank, 2018) and;
VI. It is likely that ongoing sensory experience would contaminate, and be contaminated by, consolidation occurring during wakefulness, suggesting this process best occur during sleep (Gais and Born, 2004a,b; Chokroverty, 2017).

Thus, even if waking cortical activity is strong enough to stand-in for NREMS oscillatory couplings and consolidate information overcoming the cholinergic repression on hippocampal-neocortical dialog it is unlikely that waking SPW-Rs serve this function. More likely, is that systems consolidation depends primarily on interacting oscillations during NREMS (Ladenbauer et al., 2017; Latchoumane et al., 2017). In which case, a cellular mechanism connecting NREMS oscillatory interactions to memory consolidation is needed (Clemens et al., 2007; Wei et al., 2016).

### A Cellular Mechanism for Systems Memory Consolidation

**Figure 1:**
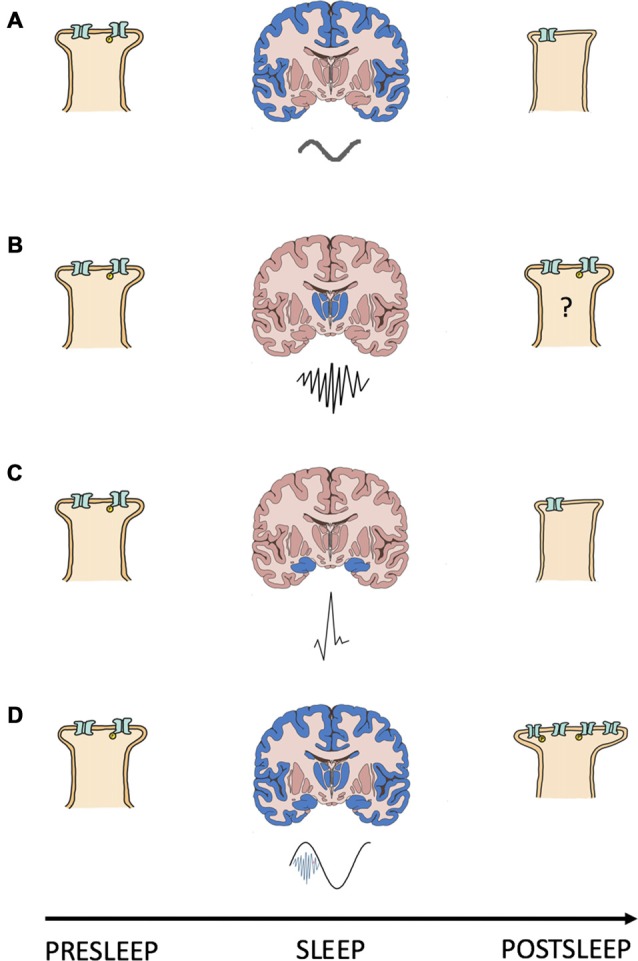
Interacting oscillations can cause the synaptic change required for NREMS systems consolidation. Shown is a schematic summary of a model for the relationship between NREMS oscillations and synapse strength changes. **(A–D)** Each panel depicts a presleep cortical postsynapse (left), a coronal brain section (middle) with the region of origin (blue) and waveform (below) of a NREMS rhythm, and the same postsynapse postsleep (right); **(A)** slow oscillation; **(B)** sleep spindle; **(C)** sharp wave and; **(D)** slow oscillation (black) coupled to a sleep spindle (blue) with a trough nested sharp wave (red). Synapse strength is indicated by the size of the postsynapse as well as by the number and phosphorylation (yellow “P”) status of receptors.

The synaptic change underlying memory storage requires the upstream activation of dendritic signaling cascades, initiated by high magnitude calcium transients, for its instatement (Ismailov et al., 2004; Yamanaka et al., 2017). Problematically, the properties of both SPWs and SOs suggest that these rhythms produce low magnitude calcium transients and synaptic depression or LTD (Tononi and Cirelli, 2006; Norimoto et al., 2018). Therefore, a mechanism operating during NREMS to augment or prolong calcium influx and mobilize potentiating cascades is required. The temporal coupling of NREMS SPW-R, SO and SS rhythms can satisfy this requirement by spatially and/or temporally summating on dendrites to: (I) increase depolarization magnitude and the number of conducting calcium channels; and/or (II) prolong depolarization duration and calcium channel open time. In either case, coupling NREMS oscillations ensures that calcium levels surpass the threshold for engaging the coupling force of STDP and consolidating memories; a summary of this model is included as Figure 1.

The known properties of NREMS oscillatory interactions are compatible with those suggested as being necessary for modifying calcium dynamics and consolidating/interregionally re-locating memories. SPW-Rs often occur during the down-up state transitions of neocortical SOs (Battaglia et al., 2004). By reaching the cortex during the depolarizing portion of the SO signaling pathways initiated by the SPW-Rs can be augmented and prolonged. Thus, by coupling with the depolarizing phase of SOs SPW-Rs may increase the number, or prolong the conductance, of membrane calcium channels, changing calcium’s kinetic profile in favor of higher dendritic concentrations and, in turn, consolidative plasticity. In support of this, Batterink et al. (2016) show that the presentation of cues associated with prior learning around the time of SO up state onset resulted in better post sleep memory performance than if cue presentation occurred around the time of SO down states.

SSs, when evoked by corticothalamic volleys triggered by SOs (Contreras and Steriade, 1995; Mölle et al., 2011), nest ripples in their troughs and in doing so transfer information about reactivated memories from the hippocampus to the cortex (Born and Wilhelm, 2012). Upon reaching the cortex, SSs are coupled to the depolarizing SO up state (Mölle et al., 2002). Through these cross-frequency interactions SSs could augment/prolong calcium conductance and trigger the signaling cascades required for consolidative plasticity. Seibt et al. (2017) demonstrate that SS associated sigma activity elevates dendritic calcium, providing experimental evidence that systems memory consolidation associated oscillations can evoke changes in dendritic calcium kinetics/dynamics. In sum, interaction amongst dynamical brain rhythms orchestrates membrane properties to gate the synaptic plasticity necessary for consolidative mnemonic processing during sleep.

## NREMS Oscillations and Forgetting

The role of sleep, and particularly NREMS, in the consolidation of memories is widely known (Rasch and Born, 2013). Less discussed is the clear role that NREMS plays in forgetting. Further, the same NREMS brain activity consolidating some memories may simultaneously mediate the forgetting of others. Specifically, while interactions between NREMS oscillations are capable of strengthening memory representations (Niknazar et al., 2015; Maingret et al., 2016), these same oscillations, when in isolation, appear capable of activating brain mechanisms that trigger forgetting (Colgin et al., 2004; Colgin, 2016; González-Rueda et al., 2018).

Sharp waves are synchronous waveforms generated spontaneously during NREMS (Patel, 2015), or by cued reactivation during wakefulness (Carr et al., 2011; Leonard and Hoffman, 2016), in the hippocampal CA3 field (Bazelot et al., 2016). These hippocampal rhythms reflect memory replay and when triggered during sleep mediate the cue-independent memory retrieval necessary for systems consolidation. At the same time a memories cortical trace is being strengthened by systems consolidation its hippocampal trace weakens (Norimoto et al., 2018). The properties of hippocampal sharp waves, including the low frequency of these rhythms, are compatible with those required for hippocampal trace weakening (Heynen et al., 1995; Colgin et al., 2004; Lu et al., 2017). The concomitant consolidation of cortical engrams and clearance of hippocampal engrams ensures that hippocampal synapses remain continually available to encode new experiences (Lubenov and Siapas, 2008) and prevents wasteful, unnecessary memory copies from being stored in the brain. Thus, memory remembering, or replay, associated SPWs simultaneously promote cortical trace consolidation and hippocampal trace clearance.

At the same time that sharp waves are driving the gradual clearance of cortically consolidating hippocampal engrams, SOs are acting to trim cortical memory representations. SOs are a low frequency, sinusoidal rhythm generated spontaneously in the giant pyramidal cells of cortical layer V (Neske, 2016) which, when coupled to SPW-Rs and SSs, consolidate memories into the neocortex, as discussed above. However, when SOs occur in the absence of memory reactivations (SPW-Rs) their low frequency activity depresses synapse strengths and eliminates cortical memories represented by small shifts in synaptic weight. Information encoded during wakefulness by large shifts in synapse weight, i.e., shifts capable of ensuring coordination of pre- and postsynaptic firing, might be protected from SO elimination (González-Rueda et al., 2018). Further, Sadowski et al. (2016) observed that SPW-Rs which produce coincident activity in CA3 and CA1 place cells are capable of evoking consolidative plasticity. Moreover, Norimoto et al. (2018) show that SPW-Rs strip new memory traces of unimportant details while simultaneously stabilizing other memory storing connections. Wei et al. (2018) suggest an additional mechanism by which SOs select memories for consolidation. These authors show that SOs encourage the consolidation of important (stronger) memories by promoting competition amongst reactivated traces. Competition favors strong memories, causing the reactivation, and thus consolidation, of weak traces to be attenuated. The selective clearance of information represented in weakly weighted synaptic diagrams can remove from memory incidental details, extract “gist” (i.e., what is stored strongly enough to survive sleep downscaling) and improve the signal to noise ratio (Tononi and Cirelli, 2014). The preceding sentence describes the synaptic homeostasis hypothesis (Tononi and Cirelli, 2003) which posits that sleep functions to normalize synaptic weights to a homeostatic set point through SO mediated downscaling.

As noted earlier in section “forgetting”, NREM SSs may have the capacity to influence forgetting. To reiterate, Saletin et al. (2011) looked at post-sleep performance on items participants were instructed to remember or to forget. In this experiment researchers recorded brain activity and found that post-sleep memory performance correlated with fast SS density. Still unclear however, is the mechanism through which SSs are selectively benefiting the remembering or forgetting of specific memories. A first possibility is that the brain is actively suppressing the replay of “to be forgotten” memories, rendering them unprotected from natural forgetting mechanisms, at the same time that “to be remembered” information is being replayed and stabilized by SS’s. Alternatively, the offline replay of memories that participants were instructed not to remember could be organized to occur during periods when depressive plasticity is favored, such as between SO depolarizations or SSs. A third option is that fast spindles are, *via* some unknown mechanism, facilitating the forgetting of memories that participants were instructed not to remember. These experiments by Saletin et al. (2011) can be interpreted as providing a rationale for further inquiry into whether SSs are implicated in forgetting, particularly the instructed forgetting of select memories.

### Cellular Mechanisms of Forgetting During NREMS

Neurobiologically, forgetting describes the process of disintegrating the cellular changes laid down during memory formation. Known and speculative mechanisms for how SPW-Rs, SOs and SSs mediate this disintegration are described here.

### Sharp Wave Ripples

Ripples, the component of memory reactivations transferring information between subcortical and cortical loci (Chrobak and Buzsáki, 1996; Axmacher et al., 2008), are, in addition to being a high frequency rhythm, often coupled with SO depolarizations and SSs (Staresina et al., 2015). As such, ripples are largely incompatible with a traditional STDP based forgetting mechanism. However, sleep SPW-R replay that reaches the cortex in sync with SO down states, up to down state transitions or in the absence of SSs may lead to the weakening of a memories neocortical trace. Such SPW-R, SO, SS coupling, or the lack thereof in the latter example, may serve to offset or segment oscillations in a way that provides an intentional method for disintegrating select engrams. Alternatively, such coupling may unfold in a predominately accidental or pathological manner, preventing the stabilization of reactivated information. In either case, trace weakening would presumably result from the conductance of only a small number of calcium channels and/or short NMDA channel open times, low magnitude calcium flux and the recruitment of depressive plasticity pathways.

Over the course of systems consolidation, memory replay initiations or the reverberations of reactivated cortical ensembles may overlap. Overlapping reactivations open the system up to the potential formation of unintentional associations and to competition amongst memories for circuit resources. Active competition amongst simultaneously or proximally reactivated memories for resources makes proactive/retroactive interference from/of actively consolidating memories initiated prior to, or following the reactivation of, a given memory possible. Overlap of this kind may be an exception and not the rule as work by Antony et al. (2018) suggests that rhythmically occurring SSs may segment memory reactivations, preventing false associations as well as pro- and retroactive interference. Analogously, memories relocating from hippocampal to cortical neuronal circuitries may contaminate or replace previously consolidated information by retroactively interfering with established cortical engram circuits. However, this, as well as the active competition scenario, is unlikely given that the cortex has nearly twenty billion neurons (Herculano-Houzel, 2009), each of which has thousands of synapses (Hawkins and Ahmed, 2016) serving as potential memory sites, making significant overlap of new and previously stored information improbable.

Weakly encoded memories are presumably less likely to be reactivated and reverberate less strongly when reactivated. The prior notion is supported by research showing that SPWs reverse LTP (Colgin et al., 2004) and that events are replayed less over time (Kudrimoti et al., 1999). Memories that experience little or no consolidative replay are vulnerable to “natural” forgetting mechanisms (i.e., neurogenesis-dependent over-writing, engram instability, etc., Davis and Zhong, 2018). Hippocampal memories subjected to these natural forgetting mechanisms would be activated even less frequently and strongly moving forward and at risk of undergoing complete disintegration prior to trace relocating by systems consolidation. Thus, NREMS SPW-Rs operate through various mechanisms to, intentionally or otherwise, forget part of or entire memories.

For those interested, Norimoto et al. (2018) provide an insightful characterization of several additional mechanisms used by SPW-Rs to activate LTD-like pathways and weaken hippocampal synapses. Amongst these mechanisms are homosynaptic and heterosynaptic depression pathways, which appear compatible with what is necessary for SPW-R mediated synaptic weakening (Lynch et al., 1977; Mulkey and Malenka, 1992; Norimoto et al., 2018).

### Slow Oscillations

SOs are well suited for triggering forgetting during sleep as these waves are associated with the disintegration of the biochemical substrates of memory (Tononi and Cirelli, 2006; Norimoto et al., 2018). As a property of their low frequency cortical SOs may cause brief NMDA channel open times, low levels of calcium influx and activation of LTD-like intracellular pathways. However, when a cortical SO (and perhaps a hippocampal SPW) lands on heavily weighted synapses the supra-threshold excitatory currents and moderate magnitude calcium transients necessary for the pattern completion of memory ensembles and trace maintenance are likely to ensue.

Notably, in addition to the biophysically triggered downscaling mediated by SOs a biochemically triggered downscaling, mediated by Homer1a (Hu et al., 2010), also operates during sleep (Diering et al., 2017). To some degree, the biochemically evoked Homer1a mediated synaptic downscaling is multiplicative (Hu et al., 2010) while the biophysically evoked SO mediated synaptic downscaling is selective, weakening unimportant (weaker) information while important (stronger) information is protected (González-Rueda et al., 2018; Wei et al., 2018). These two downscaling mechanisms act together to maintain information encoded in synaptic weighting diagrams while decreasing the overall network, energetic and material real estate information occupies in the brain. Importantly, while both mechanisms are sleep-dependent and function to downscale synapses the SO-mediated depotentiation is what is referred to throughout this text when referencing synaptic downscaling.

### Sleep Spindles

As noted earlier the precise role, if any, that SSs play in forgetting is unclear. The relatively high oscillatory frequency of SSs makes these rhythms unlikely to trigger depressive STDP mechanisms. However, given that SSs have been demonstrated to elicit calcium transients in dendrites (Seibt et al., 2017) it is possible that particular SS waveforms, or SSs timed to occur in isolation from SPW-R or SO depolarizations, could cause the activation of depressive biochemical cascades.

In sum, SPW-Rs, SOs and perhaps SSs realize a bi-directional memory processing function. By coupling across frequencies or presenting in isolation these rhythms selectively, and often simultaneously, organize the consolidation and erasure of region-specific or disparate information. At least part of this function is likely realized by activity driven manipulations of calcium dynamics. Thus, during NREMS we “remember” not only to activate mechanisms that preserve important information but also so that the same mechanisms can mediate the forgetting of information that the brain has deemed unnecessary.

## REMS Oscillations and Memory Consolidation

A core focus of this article is to demonstrate that fundamental, state-specific sleep rhythms are co-opted for memory protection and erasure. For this reason, the following two sections (REMS Oscillations and Memory Consolidation and REMS Oscillations and Forgetting) will be divided into two subsections: first, a subsection on REMS hippocampal theta, a fundamental REMS oscillation with a documented role in memory consolidation and disintegration and second, a subsection discussing several potential consolidative and forgetting functions of non-theta REMS activity.

### REMS Hippocampal Theta

Electrophysiologically, REMS is characterized by hippocampal theta (Spoormaker et al., 2013) and high frequency neocortical oscillations (Jing et al., 2016). The basic, repeating cycle of the hippocampal theta oscillations consists of both an electrically excitable peak and a less excitable trough. Research has demonstrated that place cells representing novel places are marked during encoding by dopamine/noradrenaline co-release from the locus coeruleus (Takeuchi et al., 2016) and, along with place cells representing locations recently traversed, are replayed during REMS at theta peaks (Poe et al., 2000). Consistent with the reactivation of recently active place cells during REMS theta peaks, Pavlides and Winson (1989) observed increases in place cell spike rate and the number of multiple spike bursts, as well as decreases in the interval between spikes within a burst, during sleep sessions that followed exploration involving the recorded cells place field. Taken together, these findings suggest that offline replay during the peak of the REMS hippocampal theta rhythm supports potentiation (Pavlides et al., 1988; Poe, 2017; Navarro-Lobato and Genzel, 2018), synaptic consolidation and the maintenance of new hippocampal memories (Genzel et al., 2017). The replay of recent memories during REMS theta peaks may protect new information whilst it undergoes the time protracted systems consolidation process.

### The Mnemonic Function of Non-theta REMS Oscillations

While hippocampal activity is synchronous and relatively slow during REMS (Brown et al., 2012), cortical activity is de-synchronous and fast (Scammell et al., 2017). The above subsection made the case that REMS hippocampal theta rhythms are best suited for dealing with memories that are undergoing or that have yet to begin, systems memory consolidation. In contrast, REMS cortical activity appears best suited for dealing with memories that have already undergone a period of systems consolidation. Memories systems consolidated into the neocortex during NREMS are marked by synaptic efficacy augmenting changes and have an increased likelihood of being reactivated by strong pattern completion during REMS. The reactivation of newly systems consolidated memories during REMS could serve several functions, including synaptic maintenance (Tononi and Cirelli, 2014) and consolidation (Diekelmann and Born, 2010), memory integration (Sterpenich et al., 2014) and the construction of new substrates for future learning (Tononi and Cirelli, 2014).

Particularly salient or emotional memories can be extraordinarily long lasting (Kuriyama et al., 2010; McGaugh, 2013), often without regular, conscious rehearsal. The intense cortical activity observed in REMS could stimulate, and as a result maintain, synapses, including those seldom engaged (Kavanau, 1997; Tononi and Cirelli, 2014). Thus, strong memory encoding followed by periodic REMS reactivations may offer a mechanism capable of explaining how potent but seldom used memories can persist for a lifetime.

During NREMS memories are systems consolidated and “filtered.” Filtering refers here to the removal of noise and, in the process, extraction of gist by NREMS SO downscaling. Once weak memories, and the unimportant details of stronger memories, are removed the brain needs a mechanism to ensure that the presumably important details that remain are not simply disintegrated in the next round of NREMS. The high frequency cortical activity of REMS can cause synaptic potentiation (Rasch and Born, 2013) or consolidation (Diekelmann and Born, 2010) which fulfills this role and stabilizes systems consolidated, gist extracted information. The REMS stabilization of cortical information that has survived NREMS homeostatic downscaling is implemented at least in part by facilitating the insertion of AMPARs (Tononi and Cirelli, 2003).

REMS acts on newly, NREMS consolidated memories to promote schematic integration and the formation of novel connections, the latter of which enables abstraction and divergent thought (Rasch and Born, 2013; Tononi and Cirelli, 2014). Further, REMS’s fast, de-synchronous activity may serve to more generally promote the construction of new synaptic connections (Tononi and Cirelli, 2014), providing substrates for the learning of new concepts or for the future association of existing concepts. In sum, following periods of NREMS REMS takes the information written into the cortex by systems consolidation and filtered by synaptic downscaling and makes it more permeant and useful.

## REMS Oscillations and Forgetting

Francis Crick, known for decoding the structure of DNA, was among the first to hypothesize a role for REM, or dream, sleep in forgetting (Crick and Mitchison, 1983). In 1983 Crick, and his colleague Murdoch Mitchison, suggested that REMS mediated forgetting occurs by a “reverse-learning mechanism,” which was later hypothesized to avoid/correct overloaded “neural nets” (Crick and Mitchison, 1995). Over the past thirty or so odd years we have gained considerable insight into Crick and Mitchison (1983, 1995) reverse-learning mechanism, which appears to involve many of same oscillatory regimes used for memory stabilization, including REMS theta rhythms.

### REMS Hippocampal Theta

**Figure 2:**
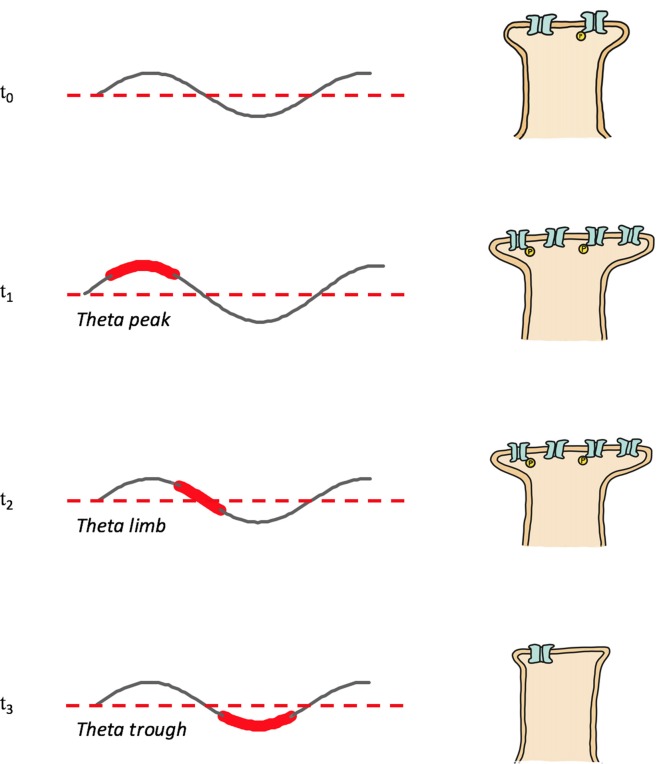
Memory replay during REMS theta shifts phases with increasing memory familiarity. Depicted are theta rhythms, theta replay phases (shown widened and in red) and synapse strengths following theta phase specific reactivations; synapse strength is represented by the size of the postsynapse as well as by the number and phosphorylation (yellow “P”) status of receptors. T0: post-encoding/pre-REMS and starting memory synapse strength; T1: theta peak replay and synapse potentiation; T2: theta limb replay, maintenance phase with no net change of synapse strength; T3: theta trough replay and depotentiation.

In addition to having a role in memory stabilization, REMS hippocampal theta mediates forgetting. Memory reactivation or replay during theta troughs causes depotentiation (Pavlides et al., 1988; Hölscher et al., 1997) and LTD (Huerta and Lisman, 1995; Hasselmo et al., 2002); presumably through pathways involving low magnitude calcium influx and the recruitment of phosphatases. Theta trough replay mediated depotentiation may be specific to connections potentiated by waking (Huerta and Lisman, 1996). In contrast to the afore described tendency for place maps encoding novel locations to be reactivated during theta peaks and strengthened (see REMS Oscillations and Memory Consolidation: REMS Hippocampal Theta), place cells representing familiar locations tend to fire during theta troughs and have their neural representations weakened, consistent with a role of REMS theta in forgetting (Poe et al., 2000; Poe, 2017); Figure 2 provides a schematic model of the memory familiarity associated theta phase shifts during replay, included also is a speculative role for theta limb replay in memory maintenance. The work of Poe et al. (2000) and Poe (2017) and others (Miyawaki and Diba, 2016) indicates that as hippocampal memories become increasingly familiar and systems consolidated the “intensity” of their replay during REMS decreases. This decrease in intensity occurs because the replay or “remembering” of memories during REMS switches from occurring during theta peaks to occurring during the less excitable theta troughs. A “memory age” dependent peak-to-trough switch allows systems consolidated memories to be cleared from the hippocampus, freeing up hippocampal substrate to be used for new learning. Consistent with a coordinated neocortical consolidation/hippocampal forgetting mechanism, Li et al. (2017) demonstrate that REMS strengthens some new cortical spines while pruning others. Thus, REMS facilitates the gradual erasure of increasingly systems consolidated hippocampal engrams by remembering these memories during theta troughs.

### The Forgetting Function of Non-theta REMS Oscillations

Several non-theta, REMS activity patterns have properties commensurate to those known to weaken memory representations. The following speculative mechanisms support the notion of an active, intentional, organized forgetting mechanism operating during REMS to not only free up space and resources but also to shape memory quality.

Following NREMS episodes, systems consolidated declarative memories are often stored in the frontal lobes (Poldrack and Gabrieli, 1997). Hypofrontality is a hallmark of REMS (Nir and Tononi, 2010), low frequency neural activity can cause subthreshold activation of neurons or weak pattern completion, and may lead to a deterioration of memories stored in this region. A mechanism of this kind would disproportionately affect weak memory representations, as strong memory representations may evoke cooperative assembly activity and be protected (González-Rueda et al., 2018). Thus, REMS may help to “fine-tune” the brains information reserves by filtering through memories newly consolidated into the frontal lobes, keeping what is strongly encoded and doing away with what is not. In this way, REMS hypofrontality could offer an algorithm for qualitatively shaping new long-term memories, extracting the gist (a function normally attributed to NREMS; Lewis et al., 2018) by trimming away the unnecessary details (Rasch and Born, 2013).

Following NREMS systems consolidation, memories exist in a sort of abyss, largely isolated from other information. In order to have the capacity for meaningful application memories often need to become integrated into various schemas (Mania et al., 2005; Schlichting and Preston, 2015). The intense activity observed over much of the cortex during REMS has been documented to serve such an information integration function (Sterpenich et al., 2014). However, integration comes at a cost. Lost in the integration process is memory “individuality.” Integrated memories run the risk of inappropriate generalization, which may cause unique attributes to be neglected if they do not match schematic “assumptions.” Further, integration has the potential to cause a blurring or loss of memory detail because of a pro/retroactive interference like overwriting of engram circuits (Runquist, 1975; Martínez et al., 2014). This type of forgetting differs qualitatively from the forgetting typically discussed and is presumptively adaptive, paving the way for the generally useful process of schematic generalization as well as for abstraction and divergent problem solving.

Waking emotional experiences are exceptionally likely to contribute to an individual’s dream narrative, this suggests emotional information is highly processing during sleep (Hobson and McCarley, 1977; Malinowski and Horton, 2014). REMS has a well-documented role in the consolidation of emotional memories (Saletin and Walker, 2012; Hutchison and Rathore, 2015). However, researchers have also shown that REMS can cause the “forgetting” of a memories emotional valence (van der Helm and Walker, 2011). Specifically, van der Helm and Walker (2011) show that REMS markers are correlated with reduced memory associated amygdalar activity and behavioral reactivity. Such REMS mediated shifts in emotional memory valence are compatible with theories suggesting that REMS functions to simulate and habituate emotional situations (Cunningham et al., 2014); see “Sleep” section. It is unclear whether the forgetting of a memories emotional charge during REMS uses hippocampal theta or other REMS oscillations, such as the lower frequency hippocampal oscillations described below.

Bódizs et al. (2001) show in humans a REMS specific, delta like SO in the hippocampus. The REMS hippocampal SO presumably serves a similar homeostatic function to the NREMS cortical SO (Tononi and Cirelli, 2014; Kim B. et al., 2017). A hypothetical model for REMS hippocampal processing, capable of integrating the presumptive function of REMS hippocampal SOs with known functions of the hippocampal theta rhythm, is as follows: REMS hippocampal theta preserves new memories by replaying them during up states and disintegrates older, already systems consolidated memories through replay on down states (Poe et al., 2000; Miyawaki and Diba, 2016; Poe, 2017). Concomitant the remembering/forgetting being carried out by theta, REMS hippocampal SO could cause a proportional decrease in synapse weights, where across synapses the magnitude of weakening is a constant proportion of synapse strength. A proportional decrease ensures that important information contained in synapse weight ratios is maintained and that synapses avoid reaching a strength ceiling (Siddoway et al., 2014). More likely, REMS SO could selectively downscale weaker synapses, while stronger synapses are largely or entirely shielded (Tononi and Cirelli, 2014).

In brief, non-theta REMS activity may possess a seldom considered forgetting function. However, this function may act primarily to alter the quality of memories, rather than, or in addition to, operating as a conventional space clearing/resource liberation mechanism.

### Dream Amnesia as a Unique Form of Forgetting

Humans experience a rapid forgetting of dream content upon waking (Segall, 1980; Mutz and Javadi, 2017). Dream amnesia is unique in so much as it does not appear to directly result from any of the eight forms of forgetting discussed here. Before discussing how, then, our dreams are forgotten we first need to consider why we dream and why it may be important that we do not remember these dreams. A popular theory postulates that dreams are simply a byproduct, an attempt by our brain to make sense of the activity coursing through its networks during sleep (Hobson and McCarley, 1977; McCarley and Hoffman, 1981). During sleep, our brains activity reflects ongoing consolidative and integrative processing. Memory processing during sleep is biased towards the preceding day or several days’ events (Freud, 1900; van Rijn et al., 2015) and explains why our dreams tend to include elements or episodes of recent experiences (Baylor and Cavallero, 2001; Payne and Nadel, 2004). An attempt to save the content of dreams could contaminate memories undergoing consolidation during sleep, diminishing memory fidelity or creating false associations. These false associations serve as another reason for why dream amnesia is adaptive; combination of the activation synthesis hypothesis of dreaming, described above, and modern research (Hobson and McCarley, 1977; Baylor and Cavallero, 2001; Payne and Nadel, 2004; van Rijn et al., 2015) indicates that while dreams contain elements and episodes from waking experience, these elements and episodes are often assembled in unexperienced combinations and with other, seemingly random, information (Hobson et al., 1987). If these partially real, partially imagined narratives were remembered it presents an opportunity for us to erroneously bias our future behavior in light of these “false” experiences.

The transience of dream memory can be speculated to result from any one, several or all of the following mechanisms: (I) during NREMS information flow between the hippocampus and cerebral cortex is reversed relative to wakefulness, favoring consolidative outward flow as opposed to memory encoding inward flow (Hasselmo, 1999). Hippocampal-cortical information flow reversal impedes dream information from entering and being encoded by weighting plasticity in the hippocampus. Further, wiring plasticity in the cortex, where the dream narrative is constructed, is likely too slow to capture dream content. The tendency to, on occasion, remember part of or an entire dream may be explained by the generation of a dream narrative in circuits where connections between activated areas exist and weighting plasticity can occur; (II) during NREMS the brains neurochemical milieu is characterized by low neuromodulatory tone (Lee and Dan, 2012). The reduced neuronal membrane excitability associated with this state-dependent change in neuromodulation attenuates encoding likelihood by reducing the probability that plasticity pathways are evoked; (III) although high cholinergic tone during REMS causes a general increase in neuronal membrane excitability the lack of noradrenergic neuromodulation during this arousal state may bar lasting synaptic change (Becchetti and Amadeo, 2016); (IV) decreased expression of plasticity related genes (Cirelli and Tononi, 2000); (V) an incompatibility between actual brain activity programs and those necessary for the successful, dream stage (NREM vs. REMS) specific encoding and recall of dreams (Marzano et al., 2011); and (VI) the hypoactivation of the prefrontal cortex during REMS (Nir and Tononi, 2010; Jakobson et al., 2012) can explain the brevity with which dreams remain in working memory, a form of memory which depends on persistent activity in this structure (Curtis and D’Esposito, 2003), upon waking.

Thus, the inability to remember dreams, regardless of whether these sleeping narratives are intentional and adaptive or desperately pieced together by our brains, may result from forgetting mechanisms seemingly uniquely suited for this function. However, it is also possible that dream amnesia is a byproduct of offline mechanisms serving to optimize the afore described bi-directional memory processing.

## NREM-REMS Cycling Coordinates Bi-Directional Memory Processing

Sleeps temporal architecture is defined by a cycling of NREM and REMS (Vyazovskiy and Tobler, 2012). The function of individual sleep stages has been extensively studied and widely discussed, while reasons for why these sleep stages alternate has received comparatively less attention. When considered together, the literature reviewed in this article alludes to a bi-directional memory processing function of NREM-REMS cycling. A memory function of NREM-REMS cycling is supported in so far as both sleep stages promote improvements in memory while selective disruption of either impairs memory (Rasch and Born, 2013). Described below is first a notional systems and then cellular level explanation of how NREM-REMS cycling accomplishes organized, efficient, bi-directional memory processing.

At the level of the hippocampus, new, strongly encoded and older, partially disintegrated memories are spontaneous reactivated during NREMS as memory trace weakening SPWs. Upon cycling into REMS, new memories are replayed on hippocampal theta peaks which re-stabilizes these memories after their weakening by earlier NREMS SPW replay. REMS re-stabilization ensures that new memories can be replayed again during successive NREMS periods, as is required for the slow, multiple reactivation-dependent process of systems consolidation. Simultaneous to the re-stabilization of new memories by REMS theta peak replays, older memories are replayed during theta troughs. REMS trough replay of older memories aids the SPWs of the preceding NREMS period in disintegrating traces (Lubenov and Siapas, 2008; Grosmark et al., 2012) which have lasted long enough to finish the time protracted systems consolidation process and frees up hippocampal substrate for new learning.

At the level of the cortex, NREMS simultaneously begins the coding in of new memory traces through systems consolidation and the weakening of these and prior consolidated traces by homeostatic downscaling. The NREMS associated weakening of memory traces in the early stages of cortical consolidation may allow unimportant information, perhaps including information stored weakly in the hippocampus just prior to sleep and surviving long enough to undergo a round of hippocampal-cortical exchange, to be removed. The co-operation of systems consolidation and synaptic downscaling, in a temporally proximal manner, prevents memories of this nature, as well as the noisy, unnecessary details of important memories, from commandeering cortical resources and influencing behavior to an extent that is disproportional to the information’s importance; this assumes that the importance of information is manifest as a memories initial encoding strength. During REMS memories that have undergone systems consolidation and survived NREMS synaptic downscaling are stabilized and integrated with existing knowledge. Subsequent NREMS cycles will continue to systems consolidate the memories that were maintained by or survived REMS hippocampal theta peak or trough replay, respectively. Additionally, subsequent NREMS phases will downscale weak connections formed by REMS.

NREM-REMS cycling can also be speculated to serve a cellular function, operating to lay down and then stabilize memories. Much like sleep, memory progresses through two stages, cellular early- and late-LTP (E-LTP and L-LTP). E-LTP is characterized by temporary (typically less than 2 h), covalent, post-translation modifications including phosphorylation of AMPA receptors and associated proteins. Comparatively, L-LTP involves protein synthesis (Huang, 1998), cytoskeletal change (Fukazawa et al., 2003) and positive feedback loops of a known memory maintenance molecule, protein kinase M zeta (Yao et al., 2009; Kwapis and Helmstetter, 2015; Langille and Brown, 2018). A model where two, interacting, sleep stages (NREM and REMS) mediate two, interacting, cellular stages of memory (E- and L-LTP or some set of equivalent processes, described below) follows: NREMS may prime cortical engram circuits by depositing E-LTP associated synaptic efficacy modifying marks which increase and reroute activity during REMS to cause synaptic consolidation or L-LTP of these newly established engram circuits. NREMS cycles into and through REMS every ninety or so minutes (Carskadon and Dement, 2011), providing enough time for the induction of many cortical E-LTP engrams while being brief enough to ensure that memories systems consolidated at the start of the NREMS period have not yet faded. NREMS systems consolidation associated, E-LTP mediated, cortical synapse strengthening may be what distinguishes cortical memories strengthened by REMS from those weakened. Namely, strengthened synapses produce larger responses (i.e., contribute to postsynapse spiking) and activate maintenance or potentiation cascades (Watt and Desai, 2010) while weaker synapses produce smaller responses (i.e., do not contribute meaningfully to postsynapse spiking) and recruit depotentiation cascades (González-Rueda et al., 2018). Notably, in this model the NREM and REMS cascades triggering E- and L-LTP, respectively, are presumed to be products of the earlier described NREM and REMS oscillatory regimes. At present, this model is purely speculative but ought to be investigated as a potential function for sleeps ultradian rhythmicity.

An integrative NREMS E-LTP REMS L-LTP memory consolidation model aligns well with the tendency for daytime naps to be occupied by REMS (Milner and Cote, 2009) and with the finding that these daytime REMS bouts improve memory (Lau et al., 2015). Experience activates cortical areas involved in processing and perception, this activation is presumably insufficient for stable cortical memory storage or there would not be a need for systems consolidation during sleep. Perhaps, experience associated activity produces temporary, E-LTP engrams in cortical circuits which ordinarily fade during waking. If so, a REMS dominated nap, occurring mid waking period (the documented mid-day dip or siesta; Roenneberg et al., 2003), before the overarching need for homeostatic synaptic downscaling presents, is well positioned to cause REMS stabilization (L-LTP) of experience elicited E-LTP cortical engrams. Research should investigate further whether E- or L-LTP cascades are predominately active during NREM and REMS. In sum, NREMS (or waking experience) may prime cortical engram circuits to be engraved into stable, lasting memory during REMS.

Importantly, the NREMS E-LTP REMS L-LTP mechanism described here should not be taken as an “end-all-be-all” mechanism but instead as more of a place holder. A mnemonic cellular function of NREM-REMS cycling may manifest as the above described mechanism or as any one of a number of equivalent processes. That is, equivalent in so far as the general purpose of said hypothetical mechanism should be to mark circuits during NREMS to be stabilized by REMS. Several speculative mechanisms include: (I) the described NREMS E-LTP REMS L-LTP model; (II) a model in which the REMS expression of immediate early genes, triggered by NREMS oscillations (Ribeiro, 2012), finalizes consolidative processes initiated in NREMS (Dudai et al., 2015); (III) a mechanism wherein NREMS produces cortical weighting plasticity and REMS drives structural plasticity or; (IV) NREMS produces both weighting and structural plasticity at a single synapse and REMS promotes the formation of new connections between implicated neurons. Or perhaps yet, if one assumes a situation where multiples rounds of NREMS are required for consolidating a memory trace, then REMS could maintain newly instated, cortical traces until the next wave of NREMS. Regardless of the mechanism, sleeps ultradian cycle likely utilizes the bi-directional memory processing properties of NREM and REMS to produce at nights end a brain that has important information stabilized and integrated, unimportant information cleared and that is ready to learn.

## Summary

Memories are remembered during NREM (SPW) and REM (theta) sleep for two reasons operating in anti-parallel: first, to consolidate recently encoded information at the systems and/or synaptic level—perhaps through a mechanism where NREMS lays down and edits an unstable cortical trace which is then stabilized and integrated by REMS—and second, to gradually erase hippocampal memory traces as the information represented by these traces is consolidated to stable neocortical storage. Additional oscillations act to fine tune the brains memory bank during sleep by interacting with ripples to stabilize adaptive information (signal) and by acting in isolation to remove irrelevant, non-adaptive data (noise); a diagrammatic summary of the discussed sleep rhythms, their origins, impact on synapse strength and memory function is included as Table 2. Ergo, the same remembering, or replay, associated oscillations used for sleep-dependent memory consolidation may operate to simultaneously erase select memories. In this way the brain has organized, or perhaps exploited, its biology such that several rhythms in the sleeping brain have been co-opted to carry out concomitantly operating, antagonistic yet complementary, mnemonic functions. In short, during sleep we remember to forget.

**Table 2:** Sleep rhythms, memory and forgetting.

Waveform	Title	Origin	Effect on synapse strength	Function
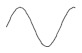	Slow oscillation	Cortex	↓	Forgetting
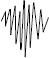	Sleep spindle	Thalamus	?	?
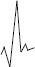	Sharp wave	Hippocampus	↑ or ↓	Forgetting*
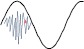	Slow oscillation, sleep spindle, sharp wave	Cortex, thalamus, hippocampus	↑	Memory
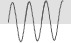	Theta (peak)	Hippocampus	↑	Memory
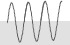	Theta (trough)	Hippocampus	↓	Forgetting
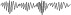	Low voltage fast (REMS)	Cortex	↑ or ↓	Memory*

Shown are NREM and REMS oscillations with the waveform, title, site of occurrence, direction of synaptic strength change and influence that each oscillation, or combination of oscillations, has on information processing indicated. In the waveform shown for the nested NREMS oscillations (slow oscillation, sleep spindle, sharp wave), a slow oscillation is indicated by black, a sleep spindle by blue and a sharp wave by red coloration. In the hippocampal theta waveforms phase is indicated by a graying of the background. *Sharp waves and low voltage fast (REMS) activity may be capable of mediating bi-directional memory processing.

## Author Contributions

JL wrote the article and designed the figures.

## Conflict of Interest Statement

The author declares that the research was conducted in the absence of any commercial or financial relationships that could be construed as a potential conflict of interest.
